# Profiling the Expression Level of a Gene from the Caspase Family in Triple-Negative Breast Cancer

**DOI:** 10.3390/ijms26157463

**Published:** 2025-08-01

**Authors:** Anna Makuch-Kocka, Janusz Kocki, Jacek Bogucki, Przemysław Kołodziej, Monika Lejman, Karolina Szalast, Anna Bogucka-Kocka

**Affiliations:** 1 Department of Pharmacology and Biology, Medical University of Lublin, 20-400 Lublin, Poland; 2 Department of Clinical Genetics, Medical University of Lublin, 20-400 Lublin, Poland; 3 Faculty of Medicine, The John Paul II Catholic University of Lublin, 20-708 Lublin, Poland; 4 Department of Biology and Genetics, Medical University of Lublin, 20-093 Lublin, Poland; 5 Independent Laboratory of Genetic Diagnostics, Medical University of Lublin, 20-093 Lublin, Poland

**Keywords:** triple-negative breast cancer, caspase family gene, apoptosis

## Abstract

It is believed that caspases may play a significant role in the development of cancer, and the expression levels of genes encoding these proteins may influence the prognosis and clinical course of cancer. Taking into account the information presented, we examined the expression profiles of 11 genes from the caspase family in patients diagnosed with triple-negative breast cancer (TNBC). We qualified 29 patients with TNBC. A fragment of the tumor and a fragment of normal tissue surrounding the tumor were collected from each patient. Then, RNA was isolated, and the reverse transcription process was performed. The expression levels of caspase family genes were determined using the real-time PCR method. The obtained data were correlated with clinical data and compared with data from the Cancer Genome Atlas database using the Breast Cancer Gene Expression Miner v4.8 and Ualcan. Based on the results of the conducted research, it can be assumed that the levels of expression of caspase family genes may be correlated with the clinical course of cancer in patients with TNBC, and further research may indicate that profiling the expression levels of these genes may be used in selecting personalized treatment methods.

## 1. Introduction

Breast cancer (BC) is one of the most frequently diagnosed cancers in women worldwide. Breast cancer is characterized by high heterogeneity caused by the influence of environmental and genetic factors [1]. Taking into account the molecular and histopathological aspects, breast cancer can be divided into several subtypes. By analyzing changes in the level of gene expression and immunohistochemical features, the expression of the estrogen receptor (ER+) or progesterone receptor (PR+), as well as that of human epidermal receptor 2 (HER2+) was determined, and triple-negative breast cancer (TNBC) was characterized by the lack of expression of the ER, PR, and HER2 [2,3]. In TNBC, due to the lack of expression of hormone receptors, hormone therapy is ineffective [4]. Triple-negative breast cancer has a severe, aggressive clinical course and is usually more common in young, premenopausal women. TNBC is characterized by rapid development of treatment resistance, poor progression, and distant metastasis as well as earlier time of local recurrence [5,6,7,8]. The exact molecular pathophysiology of triple-negative breast cancer is not fully understood; therefore it is reasonable to search for new molecular targets that enable effective treatment or earlier detection of this cancer type.

Caspases are enzymes belonging to the group of cysteine proteases. Caspases participate in the degradation of cellular proteins by cleaving peptide bonds behind the aspartic acid residue during the effector and initiator phases of apoptosis. Caspases are most often homodimers composed of two monomers. Each monomer is made of two subunits: a larger one (approx. 17–21 kDa) and a smaller one (approx. 10–13 kDa). The active site of caspases consists of five conserved loops: L1, L2, L3, L4, and L2’. Upon activation, the caspase converts to a tetramer consisting of two major subunits and two minor subunits. The caspases include initiator caspases (caspases 2, 8, 9, and 10), effector caspases (caspases 3, 6, and 7), and pro-inflammatory caspases (caspases 1, 4, 5, 11, 12, and 13). Caspase 1, 2, and 4 have a death domain and a CARD (caspase activation and recruitment domain), while caspase 8 and 10 have a DED (death effector domain). In cells that do not undergo apoptosis, caspases are in an inactive form. Initiator caspases are activated by activating complexes: DISC (death inducing signaling complex), which activates caspases 10 and 8, as well as apoptosome-activating caspase 9, and PIDDosome, which activates caspase 2. Effector caspases are in turn activated by initiator caspases [9,10,11,12,13].

Caspases are the main enzymes that regulate apoptosis and inflammation, and they maintain body homeostasis by regulating cell death. Caspases may also participate in the process of carcinogenesis and tumor development [14,15,16].

Considering the possible significant influence of caspases on the onset, course, and prognosis of cancer, the aim of our study is to evaluate changes in the level of gene expression of the caspase family in cancer tissue obtained from patients with triple-negative breast cancer compared to normal tissue. The paper presents the results of the expression levels for 11 genes from the caspase family (*CASP1, CASP2, CASP3, CASP4, CASP5, CASP6, CASP7, CASP8, CASP9, CASP10,* and *CASP14*), which were then correlated with clinical data. The obtained research results were compared with statistical data obtained from the Cancer Genome Atlas (TCGA) database using the bioinformatics tools Breast Cancer Gene Expression Miner v4.8 (bc -GenExMiner v4.8) and Ualcan.

## 2. Results

### 2.1. Determination of the Expression Levels of Genes from the Caspase Family in Patients Diagnosed with Triple-Negative Breast Cancer. Comparative Analysis with the Expression Levels of the Discussed Genes Obtained from the TCGA Database

**Figure 1:**
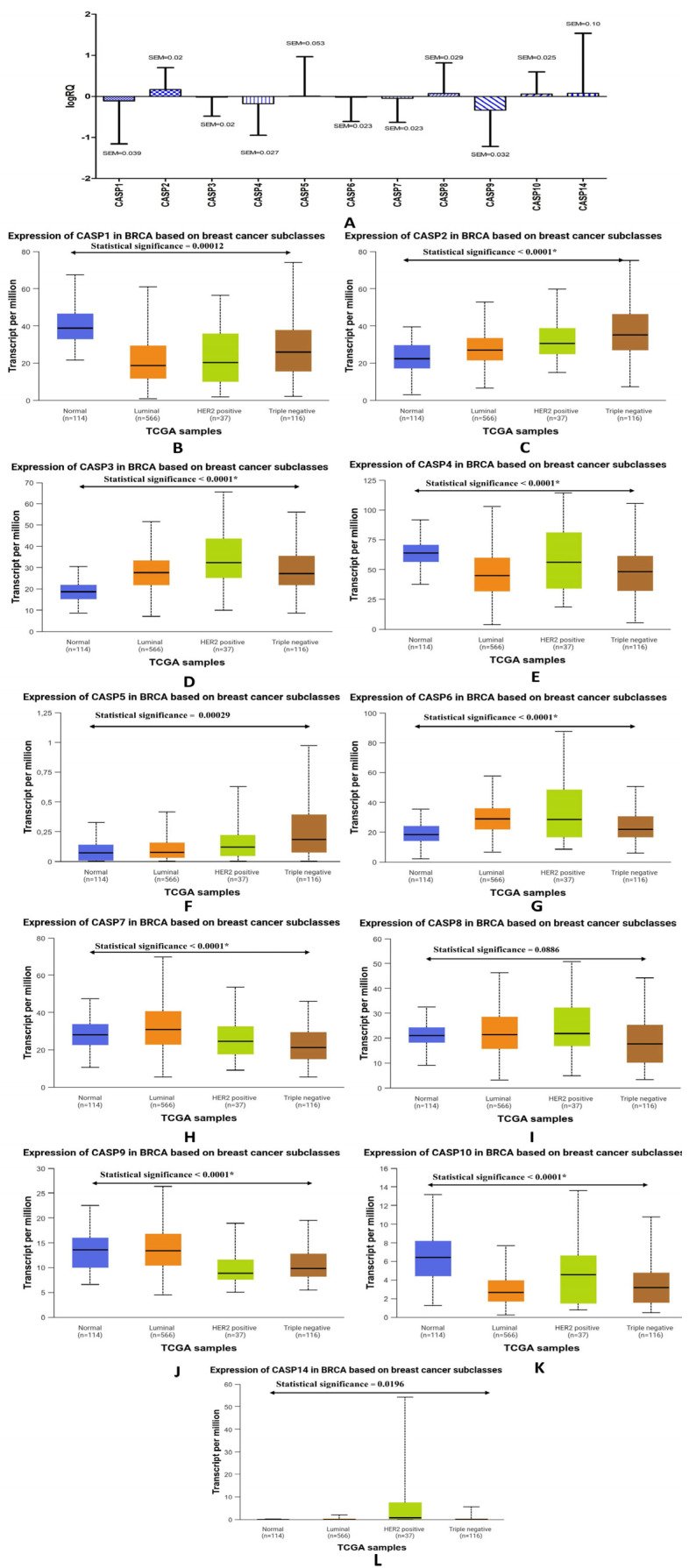
Determination of the mean level of gene expression of the caspase family in the tumor tissue of patients with triple-negative breast cancer compared to the control tissue (determined experimentally) (**A**) and comparison of the gene expression levels of the *CASP1* (**B**), *CASP2* (**C**), *CASP3* (**D**), *CASP4* (**E**), *CASP5* (**F**), *CASP6* (**G**), *CASP7* (**H**), *CASP8* (**I**), *CASP9* (**J**), *CASP10* (**K**), and *CASP14* genes (**L**) in breast cancer patients compared to normal tissue—data obtained using the Ualcan web tool from the TCGA database. Statistically significant values—*.

Figure 1A shows the expression levels of caspase family genes in tumor tissue from triple-negative breast cancer patients compared to the control tissue (non-cancerous tissue surrounding the tumor). The highest average expression level was noted for the *CASP2* gene (M = 0.168929), while the lowest average expression level was found for the *CASP9* gene (M = −0.334912). For the *CASP1, CASP3, CASP4, CASP6, CASP7,* and *CASP9* genes, a decrease in the average level of expression in the tumor tissue was noted compared to the control tissue, while the *CASP2, CASP5, CASP8, CASP10,* and *CASP14* genes showed an increase in the average level of expression in the tumor tissue compared to the control tissue control. The exact values of the level of average expression of genes from the caspase family and the parameters related to them are presented in Table 1.

The analysis of statistical data obtained from the TCGA database confirmed a statistically significant decrease in the average levels of gene expression for *CASP4, CASP7,* and *CASP9* (Figure 1E,H,J) and an increase in the average levels of gene expression for *CASP2* (Figure 1C). In contrast to the average levels of expression of the *CASP3* and *CASP6* genes (Figure 1D,G) obtained by us experimentally, the data obtained from the TCGA database indicated a statistically significant increase in the expression of these genes, while for the *CASP10* gene (Figure 1K) in the database, a statistically significant decrease in the level of expression was detected in patients with breast cancer in comparison to normal tissue. We suppose that discrepancies in the comparison of some results may result from the diversity of patients qualified for the study and the size of the experimental groups.

### 2.2. Determination of the Relationship Between the Genes of the Caspase Family. Comparison of the Results Obtained from the Experiments with the Results from the TCGA Database

**Figure 2:**
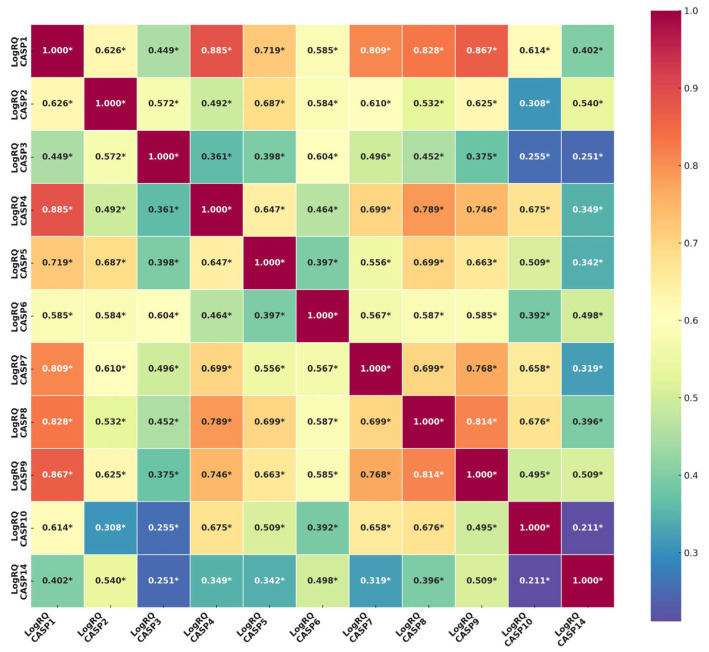
Correlation analysis of the mean expression values of genes from the caspase family in patients with triple-negative breast cancer. All obtained correlation levels are statistically significant (*—statistical significance).

Based on the statistical correlation analysis of the mean values of expression of genes from the caspase family, it can be concluded that there is a statistically significant positive correlation between all genes included in the experiments. The strongest positive correlations occur between the following genes: *CASP1* and *CASP2* (r = 0.626), *CASP1* and *CASP4* (r = 0.885), *CASP1* and *CASP5* (r = 0.719), *CASP1* and *CASP7* (r = 0.809), *CASP1* and *CASP8* (r = 0.828), *CASP1* and *CASP9* (r = 0.867), *CASP1* and *CASP10* (r = 0.614), *CASP2* and *CASP5* (r = 0.687), *CASP2* and *CASP9* (r = 0.625), *CASP3* and *CASP6* (r = 0.604), *CASP4* and *CASP7* (r = 0.699), *CASP4* and *CASP8* (r = 0.789), *CASP4* and *CASP9* (r = 0.746), *CASP4* and *CASP10* (r = 0.675), *CASP5* and *CASP8* (r = 0.699), *CASP5* and *CASP9* (r = 0.663), *CASP7* and *CASP8* (r = 0.699), *CASP7* and *CASP9* (r = 0.768), *CASP7* and *CASP10* (r = 0.658), *CASP8* and *CASP9* (r = 0.814), and *CASP8* and *CASP10* (r = 0.676). The lowest values of the correlation coefficient were found for the *CASP3* and *CASP10* genes (r = 0.255) and *CASP10* and *CASP14* (r = 0.211) (Figure 2).

**Figure 3:**
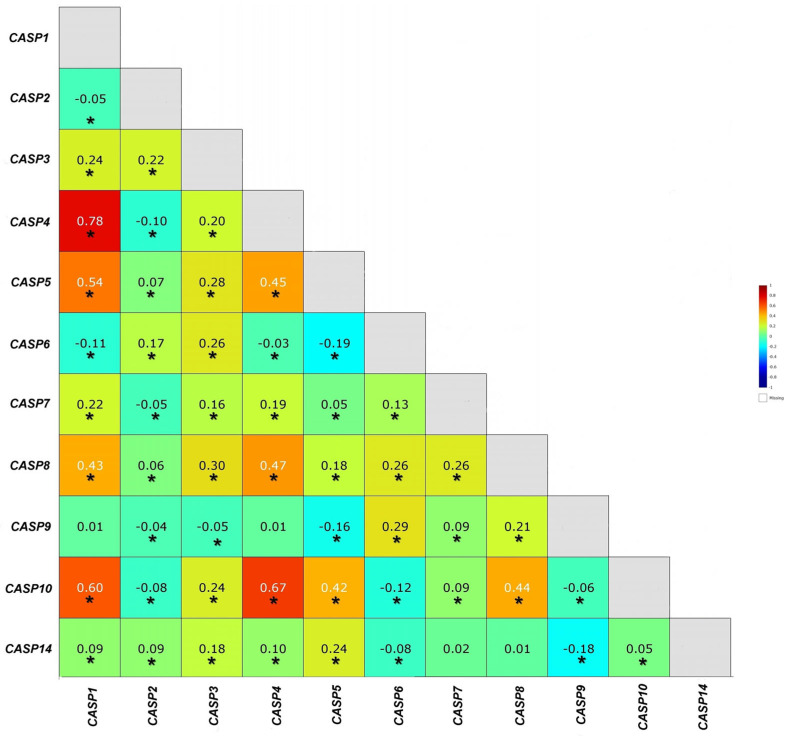
Correlation analysis of the mean values of expression of genes from the caspase family in breast cancer patients obtained from the TCGA database using the online tool Breast Cancer Gene Expression Miner v4.8 (r—Pearson’s correlation coefficient). *—statistical significance.

The analysis of statistical data contained in the TCGA database confirmed statistically significant positive correlations between experimentally determined genes, except for the following gene pairs: *CASP4* and *CASP9, CASP1* and *CASP2, CASP2* and *CASP9*, and *CASP5* and *CASP9* (Figure 3).

### 2.3. Analysis of the Relationship Between the Average Levels of Expression of Selected Genes from the Caspase Family and Clinical Data

#### 2.3.1. Age

**Figure 4:**
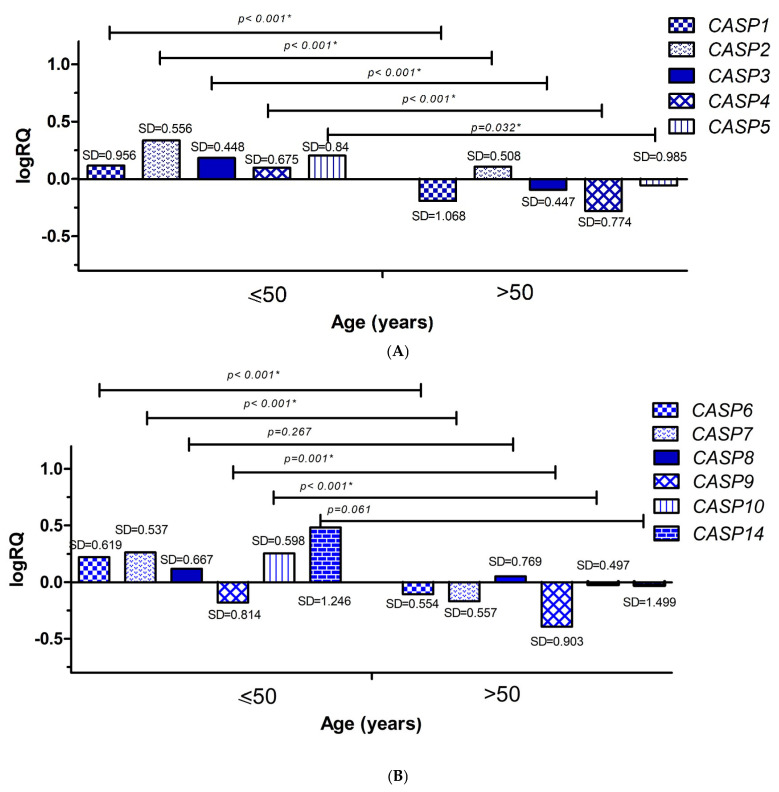
Mean expression levels (logRQ) of the *CASP1, CASP2, CASP3, CASP4*, and *CASP5* genes. (**A**) The *CASP6, CASP7, CASP8, CASP9, CASP10*, and *CASP14* (**B**) genes in the tumor tissue of patients with triple-negative breast cancer compared to normal tissue in the other age groups (≤50 years and >50 years). Values are presented as logRQ, representing the relative gene expression in tumor tissue compared to matched adjacent non-cancerous tissue (used as the calibrator). *—significance level of the Mann–Whitney U test.

In order to compare the average levels of expression of the examined genes in patients depending on the age range, patients were divided into two groups: group 1—patients who were 50 years old or younger at the time of cancer detection (n = 10) and group 2—patients who were over 50 years old (n = 19) at the time of cancer detection. Statistical analysis was performed using the Mann–Whitney U test. It was shown that the average levels of gene expression for *CASP1* (*p* < 0.001), *CASP2* (*p* < 0.001), *CASP3* (*p* < 0.001), *CASP4* (*p* < 0.001), *CASP5* (*p* < 0.001), *CASP6* (*p* < 0.001), *CASP7* (*p* < 0.001), *CASP9* (*p* < 0.001), and *CASP10* (*p* < 0.001) were statistically significantly higher in patients aged 50 years or less (Figure 4A,B).

Statistical analysis of data obtained from the TCGA database on the levels of expression of genes from the caspase family depending on the age of patients at the time of breast cancer diagnosis confirmed statistically significant higher average levels of *CASP2* (*p* = 0.0038) and *CASP4* (*p* = 0.0092) gene expression in patients aged 51 and younger compared to older people. The mean expression levels of the other genes from the caspase family did not show statistically significant differences depending on the age of the patients (Figure S1).

#### 2.3.2. Lymphovascular Invasion

**Figure 5:**
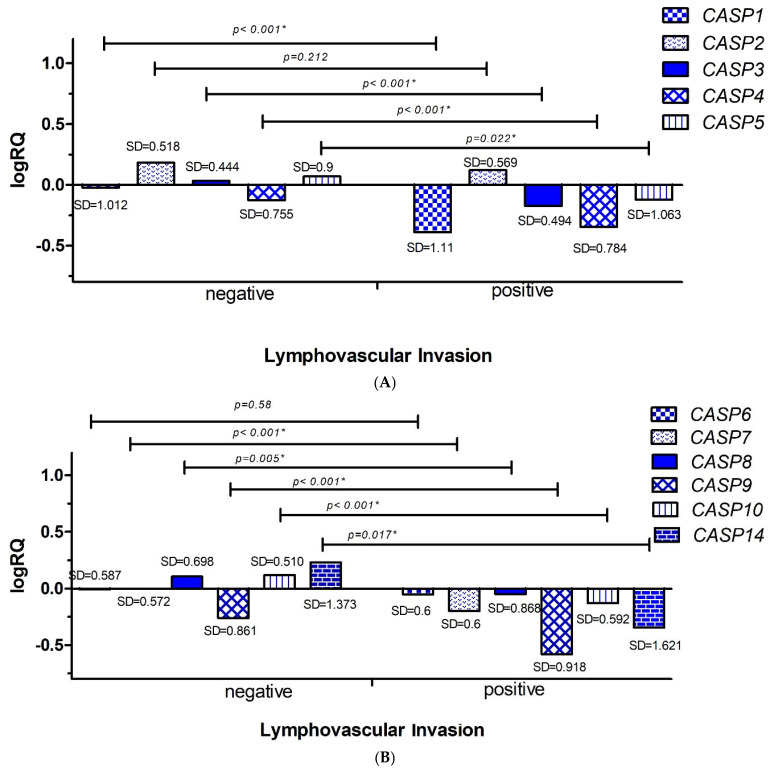
Mean expression levels (logRQ) of the *CASP1, CASP2, CASP3, CASP4*, and *CASP5* genes. (**A**) The *CASP6, CASP7, CASP8, CASP9, CASP10*, and *CASP14* (**B**) genes in the tumor tissue of patients with triple-negative breast cancer compared to normal tissue in groups based on the presence or absence of lymphovascular invasion. Values are presented as logRQ, representing the relative gene expression in tumor tissue compared to matched adjacent non-cancerous tissue (used as the calibrator). *—significance level of the Mann–Whitney U test.

In the next step, the average levels of expression of the examined genes in patients were compared depending on the presence or absence of metastases to the lymphatic vessels. Statistical analysis was performed using the Mann–Whitney U test. It was shown that the average levels of gene expression for *CASP1* (*p* < 0.001), *CASP3* (*p* < 0.001), *CASP4* (*p* < 0.001), *CASP5* (*p* = 0.022), *CASP7* (*p* < 0.001), *CASP8* (*p* = 0.005), *CASP9* (*p*< 0.001), *CASP10* (*p* < 0.001), and *CASP14* (*p* = 0.017) were statistically significantly higher in patients with undetermined invasion of neoplastic cells into the lymphatic vessels (n = 22) than in patients with the presence of neoplastic cells in the lymphatic vessels (n = 7) (Figure 5A,B).

After analyzing the statistical data obtained from the TCGA database, a statistically significant increase in the mean level of expression of the *CASP5* gene was found in patients without lymphovascular invasion (Figure S2).

#### 2.3.3. Invasion of the Fat Tissue

**Figure 6:**
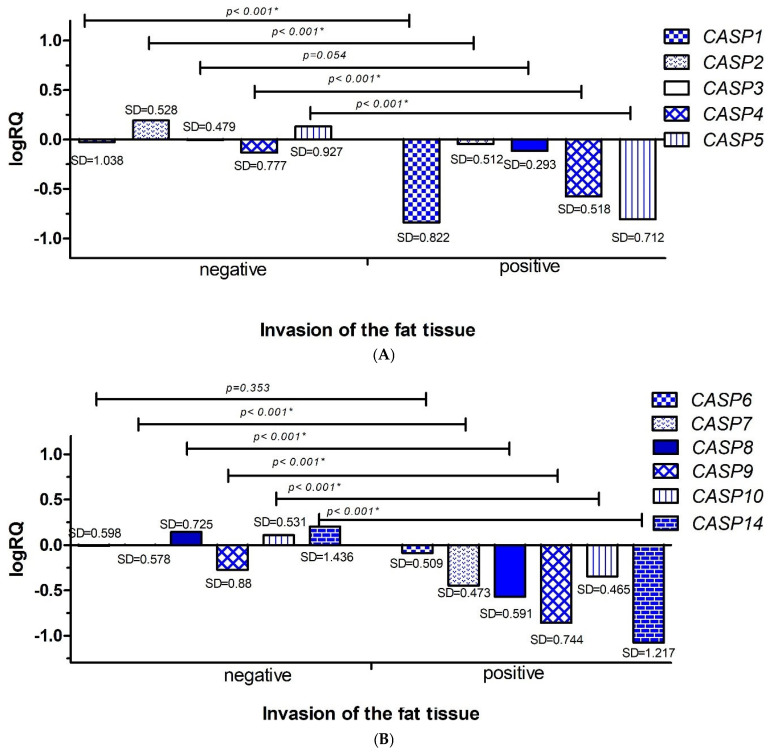
Mean expression levels (logRQ) of the *CASP1, CASP2, CASP3, CASP4*, and *CASP5* genes. (**A**) The *CASP6, CASP7, CASP8, CASP9, CASP10*, and *CASP14* (**B**) genes in the tumor tissue of patients with triple-negative breast cancer compared to normal tissue in groups based on the presence or absence of cancers cells in the fat tissue. Values are presented as logRQ, representing the relative gene expression in tumor tissue compared to matched adjacent non-cancerous tissue (used as the calibrator). *—significance level of the Mann–Whitney U test.

In the next step, the average levels of expression of the examined genes in patients were compared depending on the presence or absence of cancers cells in the fat tissue. Statistical analysis was performed using the Mann–Whitney U test. It was shown that the average levels of gene expression for *CASP1* (*p* < 0.001), *CASP2* (*p* < 0.001), *CASP4* (*p* < 0.001), *CASP5* (*p* < 0.001), *CASP7* (*p* < 0.001), *CASP8* (*p* < 0.001), *CASP9* (*p* < 0.001), *CASP10* (*p* < 0.001), and *CASP14* (*p* < 0.001) were statistically significantly higher in patients without invasion of cancer cells into the fat tissue (n = 26) than in patients with the presence of cancer cells in the fat tissue (n = 3) (Figure 6A,B).

The experimentally obtained mean expression levels of genes from the caspase family in patients with triple-negative breast cancer, depending on the presence or absence of cancer cells in fat tissue, cannot be compared with the statistical data from the TCGA database because no information on this subject was found in the database.

#### 2.3.4. The Scarff–Bloom–Richardson (SBR) Grading System

**Figure 7:**
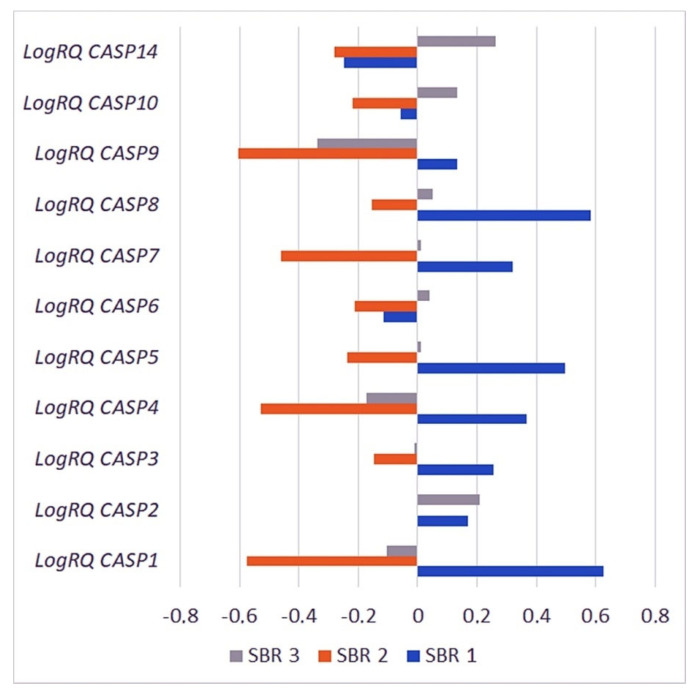
Mean expression levels of caspase family genes in patients assigned to three groups: SBR1, SBR2, and SBR3. The criterion for tumor advancement was the Scarff–Bloom–Richardson (SBR) scale. Values are presented as logRQ, representing the relative gene expression in tumor tissue compared to matched adjacent non-cancerous tissue (used as the calibrator).

The Kruskal–Wallis H test with the analysis of multiple comparisons was used for the statistical analysis of the results including the clinical parameter—qualifying patients to the appropriate group according to The Scarff–Bloom–Richardson (SBR) grading system (grade I (n = 3), grade II (n = 5), and grade III (n = 21)). Statistically significant differences in the expression of the *CASP1, CASP3, CASP4, CASP7, CASP8,* and *CASP9* genes were found in patients classified into the SBR1 and SBR2, SBR1 and SBR3, and SBR2 and SBR3 groups. Statistically significant differences in the expression of the *CASP5* gene were noted in patients from the SBR1 and SBR2 and SBR1 and SBR3 groups. For the *CASP2, CASP6*, and *CASP10* genes, statistically significant differences in the mean expression levels were observed in patients from the SBR2 and SBR3 groups (Figure 7, Table 2).

#### 2.3.5. Metastases to the Lymph Nodes

The Kruskal–Wallis H test with the analysis of multiple comparisons was used for the statistical analysis of the results including the clinical parameter that quantifies patients to the appropriate group according to metastases to the lymph nodes based on the seventh edition of the TNM classification of the American Joint Committee on Cancer (AJCC). As a criterion, the presence or absence of lymph node metastases was used. Patients in the pN0 group (n = 13) had no metastases to regional lymph nodes; patients in the pN1 group (n = 9) had micrometastases or metastases in 1–3 axillary lymph nodes; patients in the pN2 group (n = 5) had metastases in 4–9 axillary lymph nodes, and patients in the pN3 group (n = 2) had metastases in 10 or more axillary lymph nodes [17].

**Figure 8:**
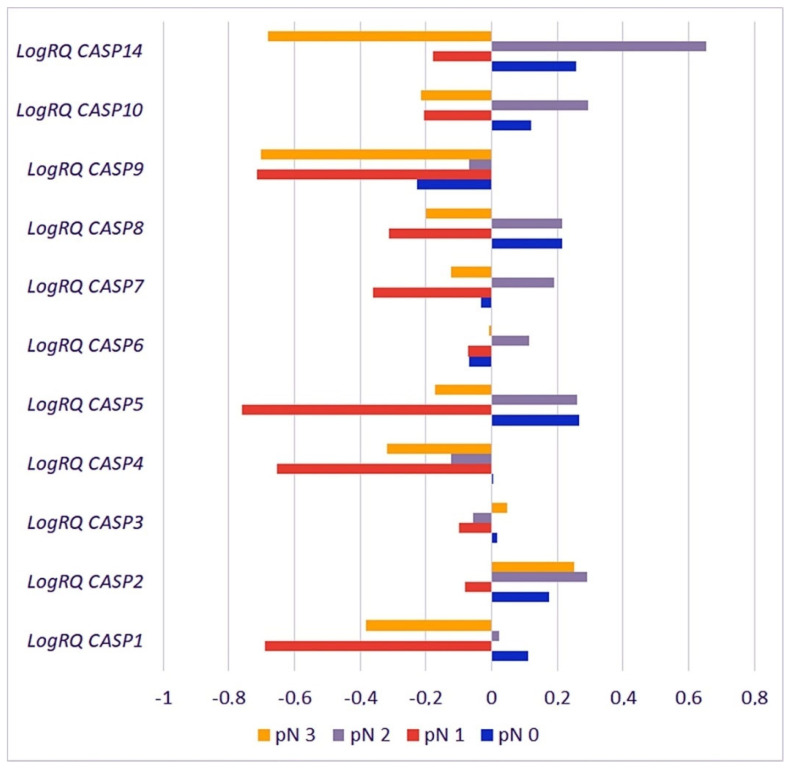
Mean expression levels of caspase family genes in patients assigned to the following four groups: pN0, pN1, pN2, and pN3. Values are presented as logRQ, representing the relative gene expression in tumor tissue compared to matched adjacent non-cancerous tissue (used as the calibrator).

Statistically significant differences in the expression of the *CASP4* and *CASP5* genes were found in patients classified into the pN0 and pN1, pN0 and pN3, pN1 and pN2, and pN1 and pN3 groups. The *CASP1, CASP8,* and *CASP9* genes showed statistically significant differences in the average expression level in groups pN0 and pN1, pN0 and pN3, pN1 and pN2, and pN2 and pN3. The mean values of *CASP2* gene expression differed statistically significantly in groups pN0 and pN1, pN1 and pN2, and pN1 and pN3; the *CASP14* gene in groups pN0 and pN3, as well as pN2 and pN3; and the *CASP6* gene in groups pN0 and pN2, as well as pN1 and pN2. For the *CASP7* gene, statistically significant differences in the mean levels of expression were found in patients classified into the groups pN0 and pN1, pN0 and pN2, pN1 and pN2, pN1 and pN3, and pN2 and pN3, while for the *CASP10* gene a statistically significant difference in expression was found in the groups pN0 and pN1, pN0 and pN2, pN0 and pN3, pN1 and pN2, and pN2 and pN3 (Figure 8, Table 3).

Statistical analysis of the data deposited in the TCGA database confirmed statistically significant differences in the level of expression of the *CASP2* gene in patients classified into the pN1 and pN3 groups and the *CASP5* gene in patients from the pN0 and pN3 and pN1 and pN3 groups. In addition, the analysis of data from the database showed statistically significant differences in the expression level of the *CASP2* gene in patients from the pN0 and pN3 and pN2 and pN3 groups, the *CASP3* gene in patients from the pN0 and pN2 and pN2 and pN3 groups, and the *CASP5* gene in patients from the pN2 and pN3 groups (Table S1).

#### 2.3.6. Primary Tumor Size

Patients participating in the study were classified into three groups depending on the size of the primary tumor based on the seventh edition of the TNM classification of the American Joint Committee on Cancer (AJCC). The primary tumor size in patients in the pT1 group (n = 3) was ≤ 20 mm; in the pT2 group (n = 19), it was > 20 mm but ≤ 50 mm, and in pT3 (n = 7), the primary tumor size was greater than 50 mm [11].

**Figure 9:**
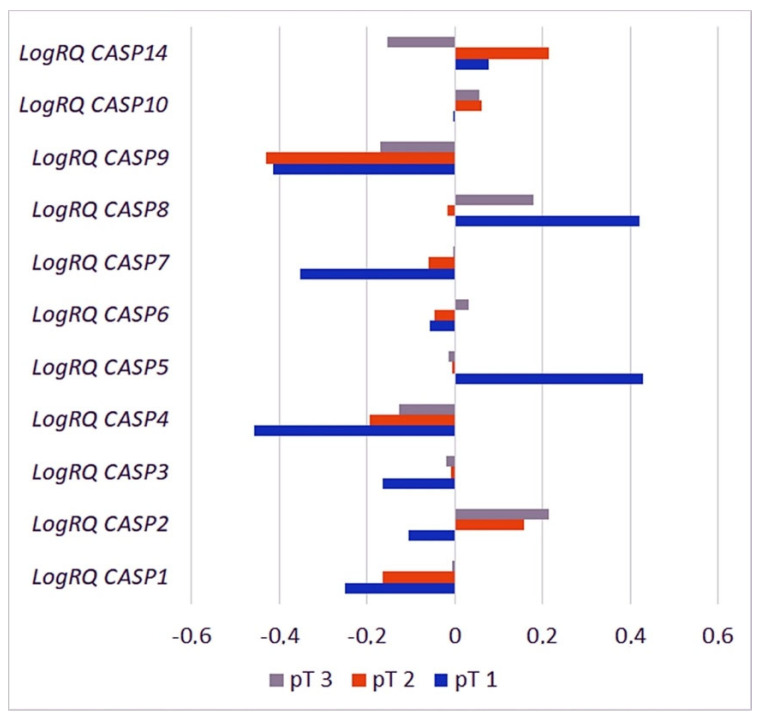
Mean expression levels of caspase family genes in patients assigned to the following three groups: pT1, pT2, and pT3. Values are presented as logRQ, representing the relative gene expression in tumor tissue compared to matched adjacent non-cancerous tissue (used as the calibrator).

The Kruskal–Wallis H test with the analysis of multiple comparisons was used for the statistical analysis of the results including the clinical parameter—qualifying patients to the appropriate group according to the primary tumor size. The analysis of the experimental data showed a statistically significant difference in the mean level of CASP2 gene expression in patients classified into the pT1 and pT3 groups, CASP7 gene expression in patients from the pT1 and pT2 and pT1 and pT3 groups, CASP8 gene expression in patients from the pT1 and pT2 and pT2 and pT3 groups, and the CASP9 gene in patients from the pT2 and pT3 groups (Figure 9, Table 4).

#### 2.3.7. Analysis of Data from the TCGA Database on the Impact of the Expression Levels of Selected Genes from the Caspase Family on the Overall Survival of TNBC Patients

**Figure 10:**
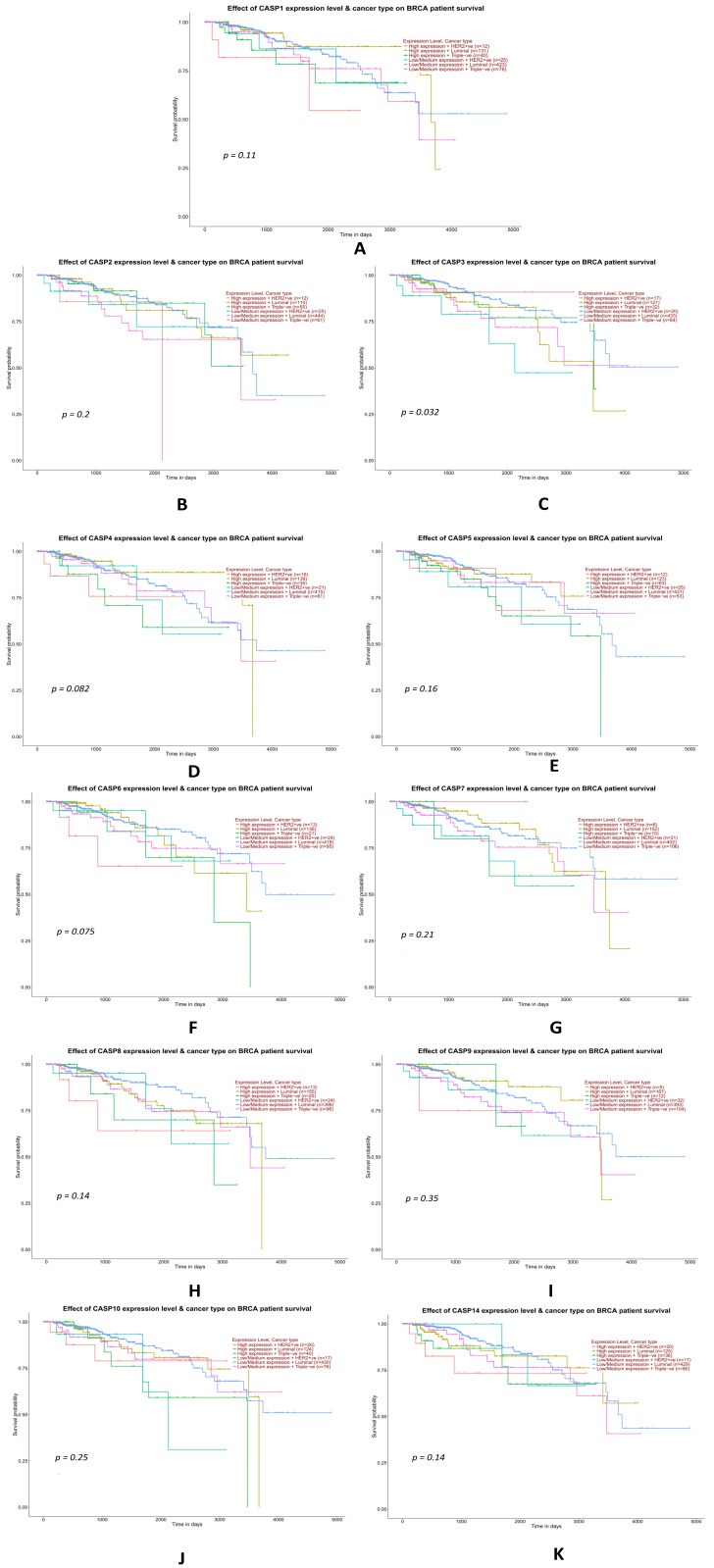
Survival curves presented using Kaplan–Meier plots showing the assessment of the prognostic significance of caspase family genes, namely *CASP1* (**A**), *CASP2* (**B**), *CASP3* (**C**), *CASP4* (**D**), *CASP5* (**E**), *CASP6* (**F**), *CASP7* (**G**), *CASP8* (**H**), *CASP9* (**I**), *CASP10* (**J**), and *CASP14* (**K**), in breast cancer patients (different molecular subtypes). Data were obtained using the online tool Ualcan. Note: Only *CASP3* (panel c) showed a statistically significant association with survival (*p* = 0.032); the other comparisons did not reach significance and are included for exploratory purposes.

Statistical analysis of data from the TCGA database using a Kaplan–Meier plotter showed a statistically significant effect of an increase in the mean level of expression of the CASP3 gene (*p* = 0.032) on the shorter survival of patients with TNBC compared to patients with other molecular subtypes of breast cancer. For the remaining genes (*CASP1, CASP2, CASP4, CASP5, CASP6, CASP7, CASP8, CASP9, CASP10,* and *CASP14*), the differences in survival between the high- and low-expression groups were not statistically significant, although the data are included for completeness and exploratory interpretation (Figure 10A,B,D–K). We do not have experimental data for this parameter (Figure 10).

Although only one gene (*CASP3*) showed a statistically significant association with overall survival (*p* = 0.032), the survival plots for other caspase genes are still informative from an exploratory standpoint. Given the complex roles of caspases in apoptosis, inflammation, and tumor progression, these trends may reflect subtle or context-dependent contributions to tumor biology. These findings, while not conclusive, support the need for further research into the prognostic and predictive utility of caspase expression in TNBC.

## 3. Discussion

Analyzing the literature data available to us, we can conclude that for the first time we have made an experimental and simultaneous assessment of the expression levels of 11 genes from the caspase family (the *CASP1, CASP2, CASP3, CASP4, CASP5, CASP6, CASP7, CASP8, CASP9, CASP10,* and *CASP14* genes) in patients diagnosed with triple-negative breast cancer. Most of the studies conducted by other authors so far have been based mainly on the use of commercially available cell lines or bioinformatics analysis of statistical data deposited in publicly available databases. When analyzing such data, it should be noted that the results contained in the databases may differ in terms of the methodology used or the conditions for classifying patients for the study, which may affect the quality and usefulness of the obtained analysis. Some of the available works on genes from the caspase family also did not consider the division of breast cancer into their molecular subtypes and the inclusion of triple-negative breast cancer as a separate subject of research. In connection with the information presented above, we have reason to believe that our research may bring important information to the world of science regarding the profiling of the levels of expression of genes from the caspase family in patients with triple-negative breast cancer, which may contribute to completing the molecular characteristics of this subtype of breast cancer.

In our work, we take into account the results of the expression levels of 11 genes from the caspase family determined in patients with TNBC. The study used homogeneous criteria for qualifying patients to the study group, and the results were obtained using a unified study methodology for each of the tested samples.

Caspases play a key role in controlling the processes of cell death and inflammation. Too low a level of activation of caspases may contribute to the development of a neoplastic process or infection. Caspases are essential for maintaining proper homeostasis of the body and are a potential target for newly developed anti-cancer therapies [18]. In view of the above, it seems reasonable to characterize the level of genes from the caspase family in patients with breast cancer.

In the first stage of the statistical analysis of the results we obtained, we observed increased levels of expression of the *CASP2, CASP5, CASP8, CASP10,* and *CASP14* genes compared to normal tissue, while the *CASP1, CASP3, CASP4, CASP6, CASP7,* and *CASP9* genes showed a decrease in the levels of expression compared to the control (Figure 1). The highest level of expression was noted for the *CASP2* gene, which encodes caspase 2 and is classified as initiator caspases; it contains the caspase recruitment domain (CARD). According to the literature, the loss of caspase 2 may increase the ability of cells to undergo neoplastic transformation, which is why it can be classified as a tumor suppressor protein. Caspase 2 may protect cells against damage to genetic material and the development of cancer [18].

Analyzing clinical data, we showed that the average levels of *CASP1, CASP2, CASP3, CASP4, CASP5, CASP6, CASP7, CASP9, CASP10* gene expression were statistically significantly higher in patients up to 50 years of age (Figure 4A,B). Taking into account the clinical data on the infiltration of cancer cells into the lymphatic vessels, we showed that the mean expression levels of the *CASP1, CASP3, CASP4, CASP5, CASP7, CASP8, CASP9, CASP10,* and *CASP14* genes were statistically significantly higher in patients without the presence of cancer cells in the lymphatic vessels (Figure 5A,B). In the case of the analysis of the levels of expression of the discussed genes and the infiltration of cancer cells into adipose tissue, we showed statistically significantly higher levels of expression of the *CASP1, CASP2, CASP4, CASP5, CASP7, CASP8, CASP9, CASP10,* and *CASP14* genes in patients without signs of neoplastic invasion in adipose tissue (Figure 6A,B).

Song and colleagues, based on a bioinformatics analysis of available statistical data from the Breast Cancer Gene-Expression Miner database, showed a high mean level of expression of the *CASP1* gene in TNBC patients. Further analysis showed that high levels of *CASP1* expression are associated with a poor prognosis in breast cancer patients and play a large role in tumor cell invasion [19]. In our experiments, the average expression level of the *CASP1* gene was decreased in the test sample compared to the control sample. We also observed a higher level of *CASP1* gene expression in patients without tumor cell infiltration into adipose tissue and without lymphovascular invasion. The differences in our results compared to Song and colleagues may result from the research methods used and the selection of patients in the study group. Our research was performed experimentally, while Song and colleagues used the analysis of statistical data from publicly available databases using bioinformatics tools.

The article by Adinew and colleagues, in which integrated bioinformatics methods were used as a research method, showed a reduced level of expression of the *CASP4* gene in patients with triple-negative breast cancer. The presented results are consistent with our experimental data, and we also noted a decrease in the mean level of expression of the *CASP4* gene in the tumor tissue of TNBC patients compared to the control tissue [16].

The work of Peng et al., using bioinformatic analysis of TCGA data on the expression level of the *CASP1* gene in patients with breast cancer, showed that the average expression level of the gene in question is lower in breast cancer tissues than in the surrounding tissues, which coincides with our experimental results. Additionally, researchers showed that patients with high average *CASP1* gene expression had better overall survival than patients with low expression levels. In our study, statistical analysis of data from the TCGA database using a Kaplan–Meier plotter did not show a statistically significant impact of an increase in the average *CASP1* gene expression level on the survival of TNBC patients. The difference may result from the selection of the study group for the analysis. We included only one molecular subtype of breast cancer—TNBC—while Peng used all breast cancer patients available in the database in the analysis [20].

Kołacińska and colleagues examined the expression level of several selected genes, including *CASP3*, using the real-time PCR method in patients with various molecular subtypes of breast cancer (including 17 patients with TNBC) not subjected to neoadjuvant chemotherapy compared to control samples taken from patients without cancer. (control group). The obtained results were compared with the pathological response of the tumor after the treatment. Statistical analysis did not show a statistically significant correlation between the level of *CASP3* gene expression and the pathological response in patients, regardless of the breast cancer subtype [21].

Based on the data available to us, we can assume that we were the first to perform an experimental, simultaneous, and comprehensive analysis of the expression level profiling of 11 caspase family genes in patients with triple-negative breast cancer who did not receive neoadjuvant chemotherapy before collecting the material for testing. However, it must be noted that our study focused exclusively on the transcriptional level of caspase family genes and did not include an assessment of their protein levels or enzymatic activities. Despite these limitations, transcriptomic profiling remains a valuable initial step in identifying potentially relevant regulatory pathways and molecular markers.

The presented results, based on a homogeneous group of TNBC patients and validated with TCGA data, reveal meaningful correlations between caspase gene expression and various clinical parameters, suggesting potential biological and prognostic significance. These findings are consistent with some previous reports at the mRNA and protein levels and support the role of caspases in TNBC pathology.

Therefore, this study should be considered a preliminary examination. It provides a valuable transcriptional framework that can guide future functional and proteomic investigations aimed at elucidating the biological roles of individual caspases in TNBC progression.

When analyzing the data we obtained, it should also be noted that the expression of individual genes from the caspase family does not show a uniform, linear relationship with all the assessed clinical and pathological parameters. The variability of caspases in relation to parameters such as age, the grade of malignancy (SBR), the presence of lymph node metastases, or fatty infiltration may result from the multifaceted role of these enzymes. Caspases participate not only in classical apoptosis but also in inflammatory processes, the regulation of the cell cycle, differentiation, and even signaling unrelated to cell death. As a result, their biological function is strongly dependent on the microenvironmental context and the stage of tumor progression [22].

The small size of the study group is related to the fact that TNBC is the rarest molecular subtype of breast cancer in the population. However, we believe that in the global context and the subject of basic research, these data make an important contribution to the field of science of profiling the expression levels of caspase family genes in TNBC. The method of applying statistical analyses and, above all, interpreting the results is typical for statistics used in groups of small numbers. Therefore, we treat the conclusions from the statistical analyses as exploratory conclusions that cannot be the basis for rigorous statements.

To sum up, based on our experiments, the analysis of statistical data from publicly available databases, and a literature analysis, it can be assumed that the expression levels of caspase family genes may be related to the clinical course and prognosis of triple-negative breast cancer. The expression levels of selected genes from the caspase family may also be related to the stage of cancer advancement and may indicate the patients’ potential chances of survival. It seems reasonable to state that the assessment of the expression profiles of caspase family genes in patients with breast cancer may constitute the basis for the search for new biomarkers supplementing the standard classification of receptor subtypes in triple-negative breast cancer in order to select appropriately personalized and effective treatment for oncological patients. To achieve this goal, further broader studies involving a larger group of patients and studies in in vitro and in vivo models are necessary.

## 4. Materials and Methods

### 4.1. Characteristics of the Study Group of Patients

The biological material used in the experiments was collected from 29 patients hospitalized in the Oncology Center of the Lublin Region who were diagnosed with triple-negative breast cancer. Prior to the study, consent from the Ethics Committee of the Medical University of Lublin was obtained (26 June 2014, decision number: KE-0254/216/2014). The tests were performed in accordance with the guidelines of the Declaration of Helsinki. Based on the information obtained from the medical interview, it was noted that the patients qualified for this study had not previously undergone chemotherapy, their family members did not have cancer, and no additional chronic diseases were found in the patients. Based on the Scarff–Bloom–Richardson score, the 4th edition of the World Health Organization (WHO) Cancer Classification for breast cancer [23], and the 7th edition of the American Joint Committee on Cancer (AJCC) TNM classification, breast cancer staging, histological type, and the presence/absence of metastases were determined based on lymph nodes (pTNM) [17,23]. The level of expression of the studied genes was also analyzed in patients divided into two groups depending on the presence of cancer cells in the adipose tissue of the mammary gland in close proximity to the tumor. The result of the histopathological examination enabled the division of patients into two groups: with the absence of cancer cells in the adipose tissue in the vicinity of the tumor and patients in whom the examination revealed that the tumor infiltrated the surrounding adipose tissue. Table 5 presents the clinical characteristics of the patients included in this study.

### 4.2. Preparation of Material for Research

The material necessary for the study, taken from the patients during surgery, was a fragment of the tumor (research sample) and a fragment of normal tissue surrounding the tumor (control sample). The histopathological evaluation of the collected materials was performed by two independent histopathologists, stating the presence (test sample) or absence (control sample) of neoplastic cells. The collected material was placed in sterile containers with RNA and then stored at −20 °C. To use the obtained biological material for experiments, the tissues were homogenized using a Precellys 24 homogenizer (Bertin-Instruments, Montigny-le-Bretonneux, France) with the Cryolys cooling option and subjected to the processes of RNA isolation and reverse transcription described later in the manuscript.

### 4.3. RNA Isolation, cDNA Reverse Transcription, and Gene Expression Analysis

The initial stage of the experiments was the isolation of RNA from material taken from patients. RNA isolation was performed using the modified method by Chomczyński and Sacchi [24]. In the next part of the experiment, the reverse transcription process was performed using the High-Capacity cDNA Reverse Transcription Kit (Applied Biosystem, Foster City, CA, USA) according to the manufacturer’s instructions. Subsequently, caspase gene expression levels were determined using a 384-well TaqMan™ Human Apoptosis Array (Applied Biosystems, Foster City, CA, USA) according to the manufacturer’s instructions. The endogenous control was 18S-Hs99999901_s1. To determine the level of gene expression, the real-time PCR method was used according to the previously described procedure [25]. The expression levels of the following genes were determined: CASP1-Hs00354836_m1, CASP2-Hs00892481_m1, CASP3-Hs00263337_m1, CASP4-Hs01031947_m1, CASP5-Hs00362072_m1, CASP6-Hs00154250_m1, CASP7-Hs 00169152_m1, CASP8-Hs01018151_m1, CASP9-Hs00154260_m1, CASP10-Hs01017902_m1, and CASP14-Hs00201637_m1.

The relative expression level of the tested gene was determined using the comparative method (ΔΔCt, comparative) [26]. This method allows us to calculate the relative difference in the expression level of the tested gene between the tested samples and the control sample. In the first stage, the threshold cycles (Ct) of the amplification reaction of the control and tested genes are determined for the tested samples and the control sample. Then, the differences between the Ct values of the PCR run on the matrix of the tested gene and the control gene (ΔCt) are calculated.

The calculations are performed at the same time for the tested and control samples using the following formula:

ΔCt (test sample) = Ct of the tested gene − Ct of the reference gene,

ΔCt (calibrator) = Ct of the tested gene − Ct of the reference gene.

In the next step, ΔΔCt is calculated as follows for each sample:

ΔΔCt = ΔCt (test sample) − ΔCt (calibrator).

The test sample is a fragment of tumor tissue collected from patients with triple-negative breast cancer, and the calibrator is a fragment of normal tissue surrounding the tumor.

In the next step, the normalized value of the relative expression level of the tested gene in the test sample compared to the calibrator is calculated using the following formula:

RQ = 2^−ΔΔCt^

The obtained results are proportional to the calibration sample. If the RQ value is 1, the expression level or the number of gene copies in the control and test samples are the same. RQ < 1 means that a higher level of expression of a given gene occurs in the control sample; when RQ > 1, this indicates a higher level of gene expression in the test sample compared to the control sample [26].

In order to obtain more readable results, the RQ values were analyzed after a logarithmic transformation to logRQ. If the logRQ value was zero, it meant the same level of gene expression or the number of gene copies in the control and test samples. If logRQ had a negative value, it meant a decrease in the level of gene expression in the tested sample compared to the control sample, and values greater than zero indicated an increase in the level of gene expression in the tested sample compared to the control sample [27].

### 4.4. Statistical Data Analysis

Statistical analysis and graphic design were performed using Statistica v.13.3, DisPlayr and GraphPad v.5.01. Statistical significance was determined at *p* < 0.05. The Mann–Whitney U test and the Kruskal–Wallis H test with multiple comparisons were used to calculate the differences in expression levels between genes. Correlation analysis was performed using Spearman’s r coefficient with the heat map correlation matrix.

Statistical data from the TCGA database were analyzed using the online sources Ualcan (http://ualcan.path.uab.edu/ (accessed on 30 August 2023)) [28] and Breast Cancer Gene-Expression Miner v4.8 (bc- GenExMiner v4.8, https://bcgenex.ico.unicancer.fr/BC-GEM/GEM-Accueil.php?js=1 (accessed on 30 August 2023))) [29,30].

## Figures and Tables

**Table 1:** Descriptive statistics for the average level of gene expression in patients from the study group.

Genes	The Descriptive Statistics		
	Mean [logRQ]	Median [logRQ]	SD [logRQ]
*CASP10*	0.057785	0.032619	0.542092
*CASP14*	0.073982	0.075004	1.464060
*CASP1*	−0.108449	−0.122647	1.046686
*CASP2*	0.168929	0.147210	0.530418
*CASP3*	−0.017374	0.003029	0.464245
*CASP4*	−0.178954	−0.219323	0.766781
*CASP5*	0.010069	0.035222	0.954855
*CASP6*	−0.018187	0.015360	0.589457
*CASP7*	−0.048439	−0.044794	0.583761
*CASP8*	0.070524	0.055187	0.743734
*CASP9*	−0.334912	−0.268816	0.884664

**Table 2:** Levels of statistically significant differences in the average levels of expression of genes from the caspase family in patients subordinated to the SBR1, SBR2, and SBR3 groups. *—significance level of the Kruskal–Wallis H test with the analysis of multiple comparisons.

Gene	SBR1		SBR2		SBR3		*p* for Multiple Comparisons
	Mean	SD	Mean	SD	Mean	SD	
*CASP1*	0.627288	1.252566	−0.57593	0.797737	−0.10253	1.007902	SBR1 * SBR2 < 0.001 * SBR1 * SBR3 = 0.000013 * SBR2 * SBR3 = 0.000011 *
*CASP2*	0.170370	0.479500	−0.00523	0.496358	0.208315	0.537377	SBR1 * SBR2 = 0.098684 SBR1 * SBR3 = 1.000000 SBR2 * SBR3 = 0.000636 *
*CASP3*	0.255295	0.552613	−0.14869	0.296613	−0.01232	0.476031	SBR1 * SBR2 = 0.000134 * SBR1 * SBR3 = 0.042713 * SBR2 * SBR3 = 0.003578*
*CASP4*	0.365875	0.931848	−0.52752	0.524590	−0.17403	0.744731	SBR1 * SBR2 < 0.001 * SBR1 * SBR3 = 0.000014 * SBR2 * SBR3 = 0.000001 *
*CASP5*	0.495700	0.945985	−0.23547	0.872046	0.010710	0.956223	SBR1 * SBR2 = 0.006246 * SBR1 * SBR3 = 0.046082 * SBR2 * SBR3 = 0.373080
*CASP6*	−0.11524	0.511974	−0.21179	0.524557	0.039048	0.603032	SBR1 * SBR2 = 0.898806 SBR1 * SBR3 = 0.178429 SBR2 * SBR3 = 0.000326 *
*CASP7*	0.320073	0.492996	−0.45854	0.410338	0.011265	0.579266	SBR1 * SBR2 < 0.001 * SBR1 * SBR3 = 0.001310 * SBR2 * SBR3 < 0.001 *
*CASP8*	0.581905	0.880059	−0.15437	0.654879	0.051903	0.711396	SBR1 * SBR2 < 0.001 * SBR1 * SBR3 = 0.000017 * SBR2 * SBR3 = 0.029074 *
*CASP9*	0.134908	1.169259	−0.60581	0.748388	−0.33741	0.842666	SBR1 * SBR2 = 0.000012 * SBR1 * SBR3 = 0.008075 * SBR2 * SBR3 = 0.006561 *
*CASP10*	−0.05715	0.332730	−0.21906	0.302225	0.134646	0.578614	SBR1 * SBR2 = 0.181732 SBR1 * SBR3 = 0.180342 SBR2 * SBR3 < 0.001 *
*CASP14*	−0.24895	1.444192	−0.28108	1.394317	0.263434	1.469308	SBR1 * SBR2 = 1.000000 SBR1 * SBR3 = 0.457148 SBR2 * SBR3 = 0.062783

No statistical data was found in the TCGA database for this clinical parameter.

**Table 3:** Levels of statistically significant differences in the average level of expression of genes from the caspase family in patients subordinated to the pN0, pN1, pN2, and pN3 groups. *—significance level of the Kruskal–Wallis H test with the analysis of multiple comparisons.

Gene	pN0		pN1		pN2		pN3		*p* for Multiple Comparisons
	Mean	SD	Mean	SD	Mean	SD	Mean	SD	
*CASP1*	0.1118	1.043590	−0.69059	0.780221	0.023440	1.069785	−0.38247	0.98516	pN0 * pN1 < 0.001 * pN0 * pN2 = 1.000000 pN0 * pN3 = 0.000826 * pN1 * pN2 < 0.001 * pN1 * pN3 = 0.097954 pN2 * pN3 = 0.031642 *
*CASP2*	0.1735	0.500488	−0.08197	0.502774	0.291364	0.493958	0.252147	0.62238	pN0 * pN1 = 0.000122 * pN0 * pN2 = 0.137078 pN0 * pN3 = 1.000000 pN1 * pN2 < 0.001 * pN1 * pN3 = 0.000256 * pN2 * pN3 = 1.000000
*CASP3*	0.0151	0.478217	−0.10083	0.370662	−0.055502	0.497965	0.048096	0.45006	pN0 * pN1 = 0.714210 pN0 * pN2 = 1.000000 pN0 * pN3 = 1.000000 pN1 * pN2 = 0.438126 pN1 * pN3 = 0.183985 pN2 * pN3 = 1.000000
*CASP4*	0.0028	0.786656	−0.65385	0.531883	−0.123283	0.777332	−0.31901	0.65038	pN0 * pN1 < 0.001 * pN0 * pN2 = 0.593256 pN0 * pN3 = 0.003190 * pN1 * pN2 < 0.001 * pN1 * pN3 = 0.000736 * pN2 * pN3 = 0.333208
*CASP5*	0.2668	0.861490	−0.75940	0.795295	0.258955	1.000320	−0.17339	0.82103	pN0 * pN1 < 0.001 * pN0 * pN2 = 1.000000 pN0 * pN3 = 0.032629 * pN1 * pN2 < 0.001 * pN1 * pN3 = 0.003011 * pN2 * pN3 = 0.245349
*CASP6*	−0.0682	0.599605	−0.07289	0.511774	0.114987	0.517253	−0.00789	0.72181	pN0 * pN1 = 1.000000 pN0 * pN2 = 0.003984 * pN0 * pN3 = 1.000000 pN1 * pN2 = 0.047924 * pN1 * pN3 = 1.000000 pN2 * pN3 = 0.321549
*CASP7*	−0.0336	0.568902	−0.36232	0.413360	0.190619	0.558199	−0.12252	0.66397	pN0 * pN1 = 0.000001 * pN0 * pN2 = 0.000330 * pN0 * pN3 = 1.000000 pN1 * pN2 < 0.001 * pN1 * pN3 = 0.009891 * pN2 * pN3 = 0.000315 *
*CASP8*	0.2136	0.776767	−0.31340	0.498986	0.213449	0.708702	−0.2005	0.70443	pN0 * pN1 < 0.001 * pN0 * pN2 = 1.000000 pN0 * pN3 = 0.000466 * pN1 * pN2 < 0.001 * pN1 * pN3 = 0.500580 pN2 * pN3 = 0.000819 *
*CASP9*	−0.2274	0.902092	−0.71541	0.702409	−0.068607	0.868688	−0.70153	0.78951	pN0 * pN1 = 0.000001 * pN0 * pN2 = 0.249799 pN0 * pN3 = 0.000073 * pN1 * pN2 < 0.001 * pN1 * pN3 = 1.000000 pN2 * pN3 < 0.001 *
*CASP10*	0.1206	0.552777	−0.20566	0.330053	0.293681	0.493669	−0.21419	0.56373	pN0 * pN1 = 0.000021 * pN0 * pN2 = 0.002295 * pN0 * pN3 = 0.002056 * pN1 * pN2 < 0.001 * pN1 * pN3 = 1.000000 pN2 * pN3 < 0.001 *
*CASP14*	0.2582	1.340609	−0.17743	1.442055	0.651824	1.555936	−0.68073	1.42050	pN0 * pN1 = 0.945887 pN0 * pN2 = 0.824875 pN0 * pN3 = 0.004822 * pN1 * pN2 = 0.113133 pN1 * pN3 = 0.956608 pN2 * pN3 = 0.000522 *

**Table 4:** Levels of statistically significant differences in the average levels of expression of genes from the caspase family in patients subordinated to the pT1, pT2, and pT3 groups. * Significance level of the Kruskal–Wallis H test with the analysis of multiple comparisons.

Gene	T1		T2		T3		*p* for Multiple Comparisons
	Mean	SD	Mean	SD	Mean	SD	
*CASP1*	−0.2498	0.7375	−0.163883	0.935099	−0.0049	1.2235	pT1 * pT2 = 1.000000 pT1 * pT3 = 1.000000 pT2 * pT3 = 0.662447
*CASP2*	−0.105558	0.4376	0.156733	0.538141	0.2138	0.5183	pT1 * pT2 = 0.054775 pT1 * pT3 = 0.011970 * pT2 * pT3 = 0.385085
*CASP3*	−0.163935	0.2853	−0.008704	0.451807	−0.0183	0.4988	pT1 * pT2 = 0.345270 pT1 * pT3 = 0.202814 pT2 * pT3 = 1.000000
*CASP4*	−0.458602	0.4964	−0.194676	0.705438	−0.1278	0.8717	pT1 * pT2 = 0.204427 pT1 * pT3 = 0.226328 pT2 * pT3 = 1.000000
*CASP5*	0.430275	0.6449	−0.005756	0.857957	−0.0148	1.1276	pT1 * pT2 = 0.155125 pT1 * pT3 = 0.078932 pT2 * pT3 = 1.000000
*CASP6*	−0.057051	0.4842	−0.047054	0.629186	0.0326	0.5262	pT1 * pT2 = 1.000000 pT1 * pT3 = 1.000000 pT2 * pT3 = 0.128775
*CASP7*	−0.351795	0.4073	−0.058370	0.551302	−0.0002	0.6445	pT1 * pT2 = 0.047008 * pT1 * pT3 = 0.017040 * pT2 * pT3 = 0.867840
*CASP8*	0.420419	0.4699	−0.016343	0.648543	0.1809	0.8764	pT1 * pT2 = 0.001782 * pT1 * pT3 = 0.067416 pT2 * pT3 = 0.008345 *
*CASP9*	−0.414304	0.7011	−0.430672	0.791872	−0.171	1.0130	pT1 * pT2 = 1.000000 pT1 * pT3 = 1.000000 pT2 * pT3 = 0.004854 *
*CASP10*	−0.001267	0.2651	0.062373	0.542842	0.0559	0.5628	pT1 * pT2 = 1.000000 pT1 * pT3 = 1.000000 pT2 * pT3 = 1.000000
*CASP14*	0.076860	1.3229	0.214341	1.386394	−0.1525	1.5893	pT1 * pT2 = 1.000000 pT1 * pT3 = 1.000000 pT2 * pT3 = 0.308856 *

The TCGA database does not have data on the average expression levels of genes from the caspase group depending on the size of the primary tumor detected in the patient.

**Table 5:** Clinical characteristics of the patients included in this study.

Characteristics	Patients with TNBC (n = 29)
Age ≤50 >50	8 (≈27.59%) 21 (≈72.41%)
Gender: Female Male	29 (100%) 0 (0%)
Lymphovascular invasion Yes No	7 (≈24.14%) 22 (≈75.86%)
Invasion of the fat tissue Yes No	3 (≈10.34%) 26 (≈89.66%)
Tumor size T1 T2 T3	3 (≈10.34%) 18 (≈62.07%) 8 (≈27.59%)
Lymph nodes N0 N1 N2 N3	16 (≈55.17%) 6 (≈20.69%) 5 (≈17.24%) 2 (≈6.9%)
SBR grade SBR I SBR II SBR III	3 (≈10.34%) 5 (≈17.24%) 21 (≈72.41%)

## Data Availability

The data that support the findings of this study are available from the corresponding author upon reasonable request.

## Supplementary Materials

The following supporting information can be downloaded at https://www.mdpi.com/article/10.3390/ijms26157463/s1.
