# Supplementary material for: Profiling the Expression Level of a Gene from the Caspase Family in Triple-Negative Breast Cancer
Source: Int J Mol Sci. 2025 Aug 1;26(15):7463. doi: 10.3390/ijms26157463 (PMC12347209; doi:10.3390/ijms26157463)
Supplement: Supplementary file 1 [file ijms-26-07463-s001.zip › Table S1.pdf]

Table S1. The level of significance of the difference in the expression of the studied genes in patients classified into pN0, pN1, pN2, pN3 groups by the metastases to the regional lymph nodes obtained from the Ualcan database. * Significance level.

Gene	p for multiple comparison
CASP1	pN0*pN1= 2.456400E-01<br>pN0*pN2= 9.469400E-01<br>pN0*pN3= 7.759200E-01<br>pN1*pN2= 4.038200E-01<br>pN1*pN3= 2.933000E-01<br>pN2*pN3= 8.631000E-01
CASP2	pN0*pN1= 5.606400E-01<br>pN0*pN2= 9.446000E-01<br>pN0*pN3= 9.560600E-04*<br>pN1*pN2= 6.231800E-01<br>pN1*pN3= 4.353200E-04*<br>pN2*pN3= 1.000350E-02*
CASP3	pN0*pN1= 1.341180E-01<br>pN0*pN2= 8.281500E-03*<br>pN0*pN3= 3.185000E-01<br>pN1*pN2= 8.788200E-02<br>pN1*pN3= 8.946200E-02<br>pN2*pN3= 7.564200E-03*
CASP4	pN0*pN1= 3.831400E-01<br>pN0*pN2= 6.229200E-01<br>pN0*pN3= 8.545400E-01<br>pN1*pN2= 2.735800E-01<br>pN1*pN3= 5.015000E-01<br>pN2*pN3= 7.997200E-01
CASP5	pN0*pN1= 2.446200E-01<br>pN0*pN2= 3.755800E-01<br>pN0*pN3= 5.36960000019349E-07*<br>pN1*pN2= 7.555200E-01<br>pN1*pN3= 9.843800E-04*<br>pN2*pN3= 1.249560E-04*
CASP6	pN0*pN1= 5.936800E-01<br>pN0*pN2= 2.084800E-01<br>pN0*pN3= 7.844400E-01<br>pN1*pN2= 1.067550E-01<br>pN1*pN3= 9.807400E-01<br>pN2*pN3= 2.949800E-01
CASP7	pN0*pN1= 3.739400E-01<br>pN0*pN2= 4.131400E-01

	pN0*pN3= 6.319600E-01<br>pN1*pN2= 8.384400E-01<br>pN1*pN3= 3.393800E-01<br>pN2*pN3= 3.148400E-01
CASP8	pN0*pN1= 2.583000E-01<br>pN0*pN2= 3.081400E-01<br>pN0*pN3= 3.984400E-01<br>pN1*pN2= 7.808000E-01<br>pN1*pN3= 1.543620E-01<br>pN2*pN3= 1.609450E-01
CASP9	pN0*pN1= 5.308200E-01<br>pN0*pN2= 9.815800E-01<br>pN0*pN3= 6.039400E-01<br>pN1*pN2= 7.078000E-01<br>pN1*pN3= 4.081000E-01<br>pN2*pN3= 6.732000E-01
CASP10	pN0*pN1= 9.606800E-01<br>pN0*pN2= 6.711600E-01<br>pN0*pN3= 7.566000E-01<br>pN1*pN2= 7.174800E-01<br>pN1*pN3= 7.313600E-01<br>pN2*pN3= 5.583800E-01
CASP14	pN0*pN1= 8.202200E-01<br>pN0*pN2= 8.594800E-01<br>pN0*pN3= 4.770000E-01<br>pN1*pN2= 6.585400E-01<br>pN1*pN3= 5.447200E-01<br>pN2*pN3= 3.820800E-01
