# Supplementary figures and images for: Profiling the Expression Level of a Gene from the Caspase Family in Triple-Negative Breast Cancer
Source: Int J Mol Sci. 2025 Aug 1;26(15):7463. doi: 10.3390/ijms26157463 (PMC12347209; doi:10.3390/ijms26157463)

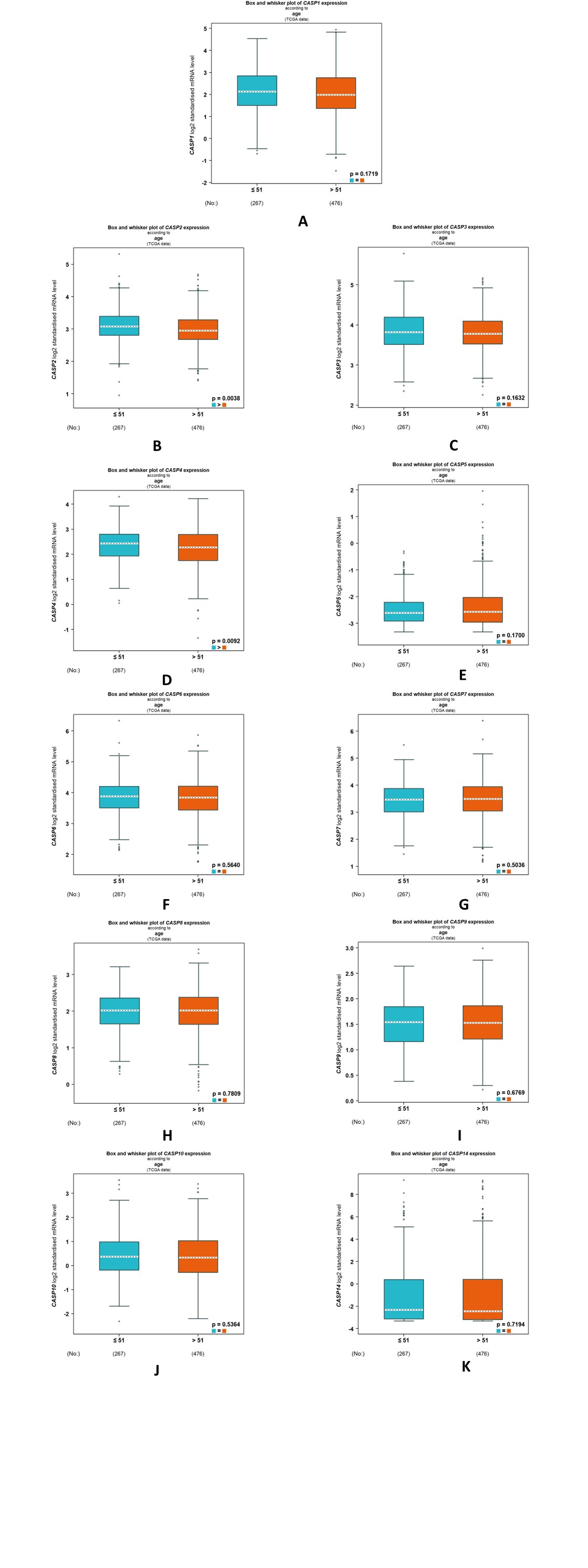

Supplement: Supplementary file 1 [file ijms-26-07463-s001.zip › Figure S1.jpg]

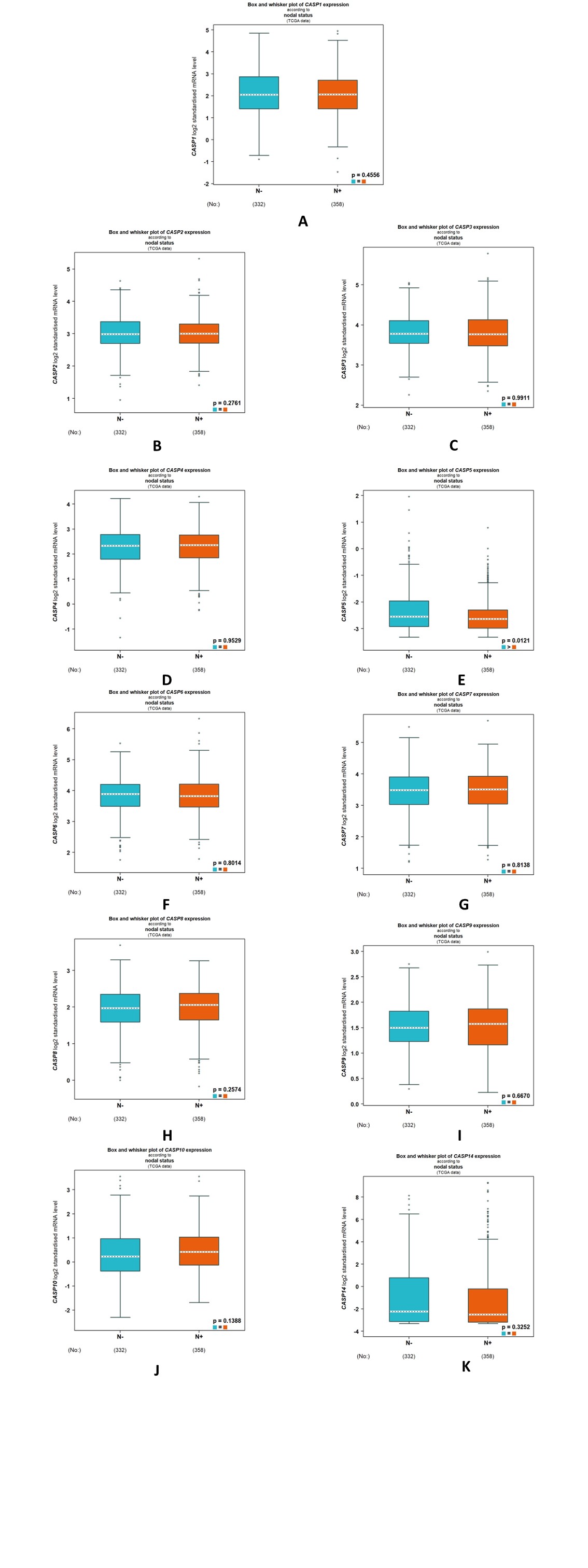

Supplement: Supplementary file 1 [file ijms-26-07463-s001.zip › Figure S2.jpg]
